# Comparative Transcriptomics of Sijung and Jumli Marshi Rice during Early Chilling Stress Imply Multiple Protective Mechanisms

**DOI:** 10.1371/journal.pone.0125385

**Published:** 2015-05-14

**Authors:** Angelica Lindlöf, Aakash Chawade, Per Sikora, Olof Olsson

**Affiliations:** 1 Systems Biology Research Centre, University of Skövde, 541 28 Skövde, Sweden; 2 CropTailor AB, Department of Pure and Applied Biochemistry, Lund University, Box 124, SE 22100 Lund, Sweden; 3 Department of Immunotechnology, Lund University, SE-22381, Lund, Sweden; 4 Department of Biological and Environmental Sciences, University of Gothenburg, SE-40530, Gothenburg, Sweden; 5 Department of Pure and Applied Biochemistry, Lund University, Box 124, SE 22100 Lund, Sweden; University of Naples Federico II, ITALY

## Abstract

**Introduction:**

Low temperature is one of the major environmental factors that adversely affect plant growth and yield. Many cereal crops from tropical regions, such as rice, are chilling sensitive and, therefore, are affected already at <10°C. Interestingly, it has been demonstrated that chilling susceptibility varies greatly among rice varieties, which indicates differences in the underlying molecular responses. Understanding these differences is vital for continued development of rational breeding and transgenic strategies for more tolerant varieties. Thus, in this study, we conducted a comparative global gene expression profiling analysis of the chilling tolerant varieties Sijung and Jumli Marshi (spp. *Japonica*) during early chilling stress (<24 h, 10°C).

**Methods and Results:**

Global gene expression experiments were conducted with Agilent Rice Gene Expression Microarray 4x44K. The analysed results showed that there was a relatively low (percentage or number) overlap in differentially expressed genes in the two varieties and that substantially more genes were up-regulated in Jumli Marshi than in Sijung but the number of down-regulated genes were higher in Sijung. In broad GO annotation terms, the activated response pathways in Sijung and Jumli Marshi were coherent, as a majority of the genes belonged to the catalytic, transcription regulator or transporter activity categories. However, a more detailed analysis revealed essential differences. For example, in Sijung, activation of calcium and phosphorylation signaling pathways, as well as of lipid transporters and exocytosis-related proteins take place very early in the stress response. Such responses can be coupled to processes aimed at strengthening the cell wall and plasma membrane against disruption. On the contrary, in Jumli Marshi, sugar production, detoxification, ROS scavenging, protection of chloroplast translation, and plausibly the activation of the jasmonic acid pathway were the very first response activities. These can instead be coupled to detoxification processes.

**Conclusions:**

Based on the results inferred from this study, we conclude that different, but overlapping, strategies are undertaken by the two varieties to cope with the chilling stress; in Sijung the initial molecular responses seem to be mainly targeted at strengthening the cell wall and plasma membrane, whereas in Jumli Marshi the protection of chloroplast translation and detoxification is prioritized.

## Introduction

Low temperature is one of the major environmental stresses that adversely affect plant growth and yield. The inherent ability of plants to endure decreasing temperatures varies extensively among species and is highly dependent on their ability to cold acclimate (which occurs at ~0–10°C) [1–3]. Many cereal crops from temperate regions, such as wheat and barley, gain increased freezing tolerance after cold acclimation, whereas tropical crops, such as rice and maize, are commonly chilling sensitive (i.e., affected already at <10°C) as they lack this ability.

Understanding the cellular and molecular responses of plants to environmental stimuli is vital for the continued development of new breeding and transgenic strategies to improve stress tolerance in crops and, consequently, these responses have been studied intensively. For example, it is now well known that cold acclimation leads to physiological and metabolic changes in both cell and tissue structures as a result of an extensive re-programming in gene expression [4–11]. A large number of genes differentially expressed during cold acclimation have been identified and characterized in the model organism *Arabidopsis thaliana* [4–11], as well as in important cold hardy cash crops like wheat and barley [12–15]. The complexity of the re-programming has also been illustrated using bioinformatics approaches, signifying that a combinatorial control of low temperature responsive genes is required to gain cold tolerance [5, 16–19].

The highly important staple food, rice, is mainly grown in warm climates (>25°C), but an exposure to chilling stress is common for rice cultivated in temperate zones or at high elevations in several places in Europe, South and Southeast Asia. Interestingly, it has been demonstrated that chilling susceptibility varies greatly among rice cultivars. For example, *Japonica* varieties generally show a greater tolerance to chilling than those of *Indica*, although variations within *Japonica* also exists [20]. Hence, it is of great scientific and economic interest to resolve underlying differences in the molecular responses to chilling stress in different varieties, identifying key regulatory components relating to these differences and infer this knowledge in the breeding programs for more tolerant varieties.

In order to gain more insights into the molecular responses, the main aim of this study was to conduct global gene expression profiling and comparative analysis of two chilling tolerant rice varieties, Jumli Marshi and Sijung (spp. *Japonica*), during early chilling stress (≤24h, 10°C). We have previously demonstrated that Jumli Marshi is more tolerant to cold stress (4°C) than the variety IR64 (spp. *Indica*) [21]. In this work, by comparative transcriptomics, similarities and differences in the genetic mechanisms underlying chilling tolerance between the two tolerant rice varieties is analyzed.

To our understanding, this is the first report on comparative global gene expression analyses of one intermediate (Jumli Marshi) and one more tolerant (Sijung) rice variety during early chilling stress. For this, the Agilent Rice Gene Expression Microarray 4x44K was used, which measures the expression level of 42,803 probes for *Oryza sativa*. The results indicate three different response strategies in Sijung and Jumli Marshi, i.e. striking differences in the number of genes differentially expressed, differences in the molecular functions of these genes and differences in the subcellular localization of the gene products. Overall, the results imply that the responses in Sijung are targeted to strengthen the cell wall and plasma membrane, whereas, in Jumli Marshi, the protection of chloroplast translation and detoxification is prioritized. Moreover, a physiological evaluation of the two varieties after 4°C treatment showed that Sijung is more cold tolerant than Jumli Marshi.

## Materials and Methods

### Plant material and growth conditions

Seeds from *Oryza sativa*, ssp. *Japonica* cv. Jumli Marshi, ssp. *Japonica* cv. Sijung and ssp. *Indica* cv. IR64 were soaked in water for 16 h at room temperature and thereafter grown on standard soil under controlled conditions, having a constant day/night temperature of 25°C and 10 hours photoperiod (250 μmol m^-2^ s^-1^ light)/14h dark cycle. 15 days after germination, seedlings were exposed to chilling stress by transferring them to cold chambers at mid-day, having the same photoperiod but an air temperature of 10°C and a light intensity of 200 μmol m^-2^ s^-1^. A slightly higher light intensity was used for regular conditions and lower intensity for stress conditions.

### Chlorophyll fluorescence measurements

Chlorophyll fluorescence was measured with the portable chlorophyll fluorometer Pocket Pea (Hansatech Instruments Ltd, UK). Plants from cv. Jumli Marshi, cv. Sijung and cv. IR64 were grown under chilling stress conditions for ten days and readings were taken at days 0, 2, 4, 6, 8 and 10 from 50 individual seedlings for each variety, and the experiment was repeated twice. Plants were dark acclimated for 1 hour before taking the measurements and the measurements were done at 10.00 pm for each time point. The photosystem II efficiencies F_v_/F_m_ = (F_m_-F_0_)/F_m_ were estimated as per the manufacturer’s instructions. Otherwise, the same growth conditions were applied to all time points and cultivars, and therefore no other recordings were made, such as ambient CO2 or air humidity, as they were not expected to vary between time points or cultivars.

### Transcript profiling

Leaf tissue from cv. Jumli Marshi and cv. Sijung plants grown under chilling stress conditions were harvested after 4 and 24 h, frozen in liquid nitrogen and stored at -80°C for further analysis. Control plants (0 h) were harvested at the beginning of the experiment, i.e., at mid-day. Total RNA was extracted from leaf tissue using TRIZOL Reagents (Invitrogen) followed by RNeasy clean-up protocol. RNA quality and concentration was measured using an Agilent 2100 bioanalyzer and Nanodrop ND-1000, respectively.

Three biological replicates were profiled for each time point (0, 4 and 24 h) and variety; hence, resulting in total 18 samples. The samples were randomly distributed and hybridized onto Agilent Rice Gene Expression Microarrays, 4x44K, containing 42,803 *Oryza sativa* probes.

In order to readily detect highly (saturated) as well as weakly (near background signal) expressed genes, scanning was done twice on the microarrays: at the PMT sensitivity level 100% (pmt100) and 10% (pmt10). The resulting images were quantified using GenePix Pro software. MIAME information describing all the 18 samples as well as raw microarray data is available at ArrayExpress (http://www.ebi.ac.uk/arrayexpress/) and are accessible through accession number E-MTAB-3121.

### Microarray data analysis

Derived GenePix (.gpr) files were processed and analyzed using methods available in the limma package for R [22]. Since each. gpr-file contains data for four arrays (i.e., 4x44K), these were split into four individual. gpr-files and thereafter imported into R. The arrays were background corrected and normalized between arrays using the normexp and quantile methods in R, respectively, and was done on all samples from both cultivars at the same time. The data distribution of the microarrays as well as the correlation between replicates were assessed after normalization and were found to be improved, i.e., there was a homogenous distribution among microarrays and a higher correlation between replicates after normalization (data not shown).

Differentially expressed genes between control and (0 h) and 4 respectively 24 h time points were assessed with linear models, using available methods in the limma package, i.e. lmFit, makeContrasts and eBayes methods [22]. The following criteria were used to filter out high quality spots and down-weighting low quality spots: Flag = 0, B532+1SD ≥ 90%, B532+2SD ≥ 90%, F532% Sat. ≤ 0.1. A Benjamini-Hochberg (BH) adjusted *P* ≤0.05 was set as significance level. Differentially expressed genes were derived from each microarray scanning (i.e., pmt10 and pmt100) separately, and duplicated genes were removed by keeping the probe with lowest BH adjusted *P* value. The resulting lists of differentially expressed genes from the two scans were thereafter concatenated.

Gene expression levels from the pmt10 scanning were used as default; the expression level from the pmt100 scanning was used only if less replicates were flagged as bad spots than from the pmt10 scanning. The expression levels from the pmt100 scanning were adjusted to the levels of the pmt10 scanning, in order to make them comparable. This was done as follows:

1. calculate the mean *m* of all expression levels for each microarray *i* and scanning, *pmt10* and *pmt100*, separately; *m*_pmt10, i = 1..18_ *and m*_pmt100, i = 1..18_
2. calculate the mean *M* of the means for the three replicates *j* at each time point *t* and scanning, *pmt10* and *pmt100*; *M*(*m*_pmt100,j = 1..3,t = 0,4,24_) and *M*(*m*_pmt10,j = 1..3,t = 0,4,24_)
3. divide *M*(*m*_pmt100,i = 1..3,t = 0,4,24_) with *M*(*m*_pmt10,i = 1..3,t = 0,4,24_) to get the scaling factor *s* for each time point *t*; *s*_t = 0,4,24_
4. divide the expression levels on each microarray from the pmt100 scanning with *s*, using the scaling factor derived for that the particular time point *t*

Clustering was performed using the Short Time-series Expression Miner (STEM) algorithm, with default parameters [23]. Pearson correlation was used to calculate the correlation between expression profiles. Fold change values were calculated by taking the average expression value of the three replicates for one timepoint in SJ and dividing it with the average expression value of the three replicates for the same timpoint in JM.

### Quantitative Real-time RTPCR measurements

TotalRNA was extracted with the RNeasy plant mini kit (Qiagen Cat. No. 74904) as per the manufacturer’s instructions. DNAse digestion was performed on-column as per the instructions using DNase Set (Qiagen, Cat. No. 79254). The primer sets for OsDreb1A, OsDreb1B, and OsDreb1C are as described in Chawade et al. (2013). Quantitative real-time RTPCR was performed on biological triplicates with iScript One-Step RT-PCR kit with SYBR Green (BioRad, Cat. No. 170–8893) using BioRad C1000 Thermo Cycler. Relative expression of the genes was calculated with the Pfaffl method (ref PMID: 11328886).

### Gene annotation analysis

Gene annotation data was downloaded from the RAP-DB database [24] and the following information was used in the analyses: Chromosomal location, Alias and InterPro signatures. GenBank IDs given for the probes on the Agilent Rice Gene Expression Microarrays were mapped to the Aliases given in the annotation file downloaded from RAP-DB.

The Singular Enrichment Analysis (SEA) tool available in agriGO [25] was used for deriving GO term annotations for up-regulated genes in Sijung and Jumli Marshi, respectively. The combined view of molecular functions and cellular components was derived by first counting how many of the up-regulated genes, for each variety separately, had been annotated with a selected molecular function and thereafter counting how many of those had been annotated with a selected cellular component. The GO annotations derived were also used for identifying various terms related to signaling and counting how many of the up-regulated genes were annotated with these terms.

For early phase responsive genes (i.e., genes up-regulated at 4 h) the RAP-DB annotation was complemented with GO annotation from the Rice Expression Profile Database (RiceXPro) [26] and for the best Arabidopsis homolog hit, as specified in RiceXPro, from the TAIR database [27]. The subcellular localization, biological process(es) and molecular function for each gene was thereafter manually inferred from the annotation. To indicate highly and weakly expressed genes, a normalized microarray signal value of 2,000 was used as discriminating level, which was arbitrarily chosen, and to indicate highly and weakly induced genes an arbitrarily chosen fold change level of 10 was used.

## Results and Analysis

### Physiological evaluation of chilling tolerance

In a previous study, we demonstrated that the variety Jumli Marshi (JM) has a higher tolerance to cold stress than IR64, since three weeks old seedlings of JM displayed a higher *F*_v_/*F*_m_ ratio during three days in cold (4°C) and was able to recover after two weeks in regular growth conditions, whereas IR64 was completely wilted [21]. The ratio between variable fluorescence (*F*_v_) and maximum fluorescence (*F*_m_) signal reflects the efficiency of Photosystem II and is an important indicator of viability [15, 28, 29]. The higher ratio in JM seedlings demonstrates that Photosystem II was less affected by the treatment than in IR64 seedlings, and consequently, was able to better cope with the stress.

**Fig 1:**
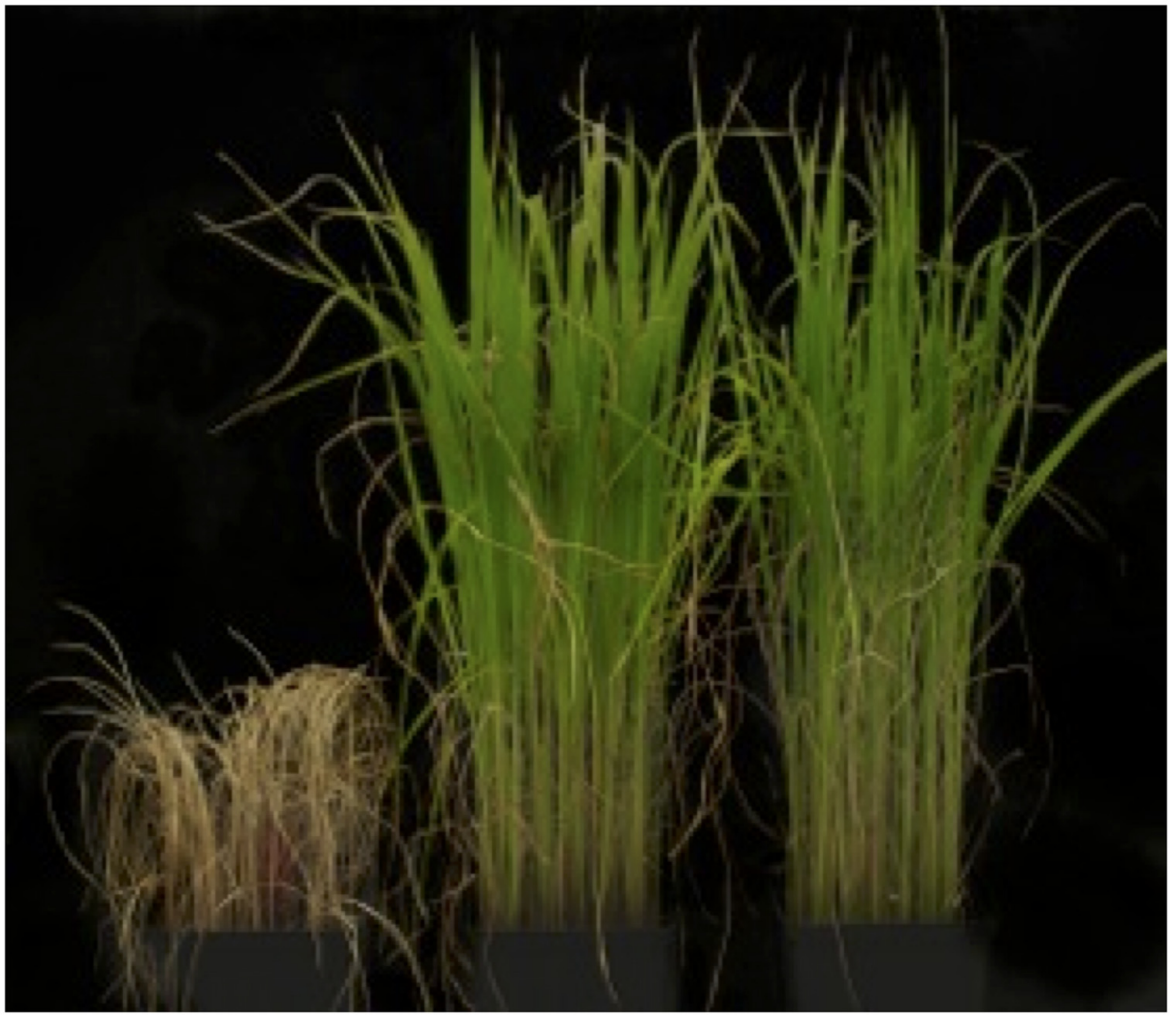
Plants exposed to chilling stress. Seedlings of IR64, Jumli Marshi and Sijung (from left to right) were grown for three weeks under regular growth conditions (a) and then moved to chilling conditions (10°C) for seven days and thereafter allowed to recover for ten days (b).

**Fig 2:**
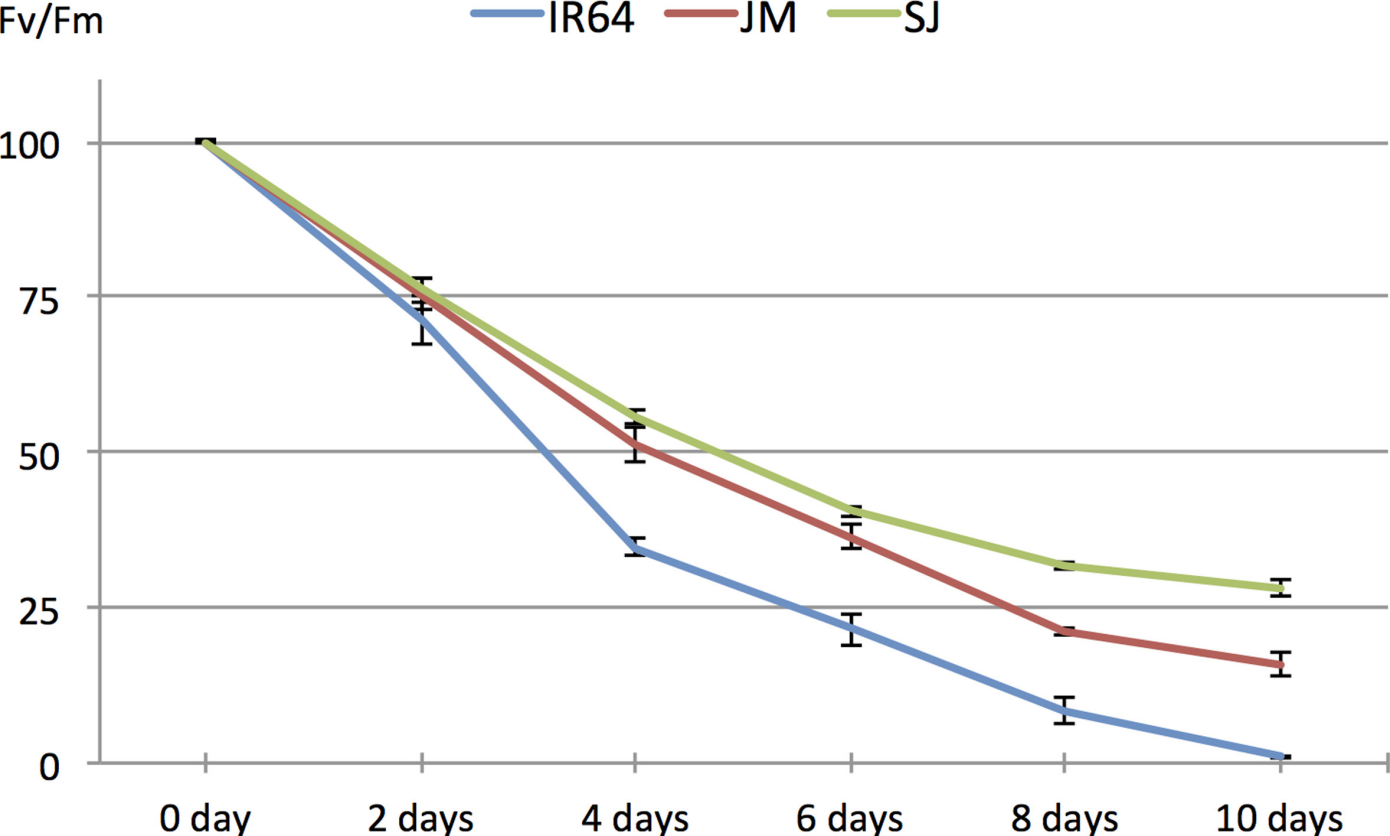
Chlorophyll fluorescence measurements. Three weeks old plants were used for the analysis. Plants were dark acclimated for 1 h and then moved to +10°C. Chlorophyll fluorescence measurements were taken from dark acclimated plants at the described time-points.

In this study, we extended the analysis to include the variety Sijung (SJ) in the comparisons, and studied early chilling stress. The physiological responses were evaluated and compared by exposing three weeks old seedlings to chilling stress (10°C) for 10 days, and thereafter allowing them to recover for two weeks in regular growth conditions (28°C) (see Materials and Methods). The stress treatment had a clear negative affect on the IR64 seedlings, as they showed complete wilting after two weeks, whereas the SJ and JM seedlings were able to survive (Fig 1). Additionally, chlorophyll fluorescence was measured at six different time points (day 0, 2, 4, 6, 8 and 10); the results showed a slightly higher tolerance by SJ compared to the other varieties, as the *F*_v_/*F*_m_ ratio was highest in this variety already after the first day (Fig 2). Moreover, it can be seen that both SJ and JM are clearly more chilling tolerant than IR64.

### Identification of chilling induced genes

The differences in the molecular responses to chilling stress in JM and SJ were elucidated using the Agilent Rice Gene Expression Microarray 4x44K. Since the interest was in the response during early chilling stress (≤24 h, 10°C), samples were harvested at three time points during this period (0, 4 and 24 h). Differentially expressed genes between control (0 h), 4h, and 24h were assessed with empirical Bayes, using available methods in the Limma package for R, and setting a Benjamini-Hochberg adjusted *P*≤0.05 as threshold [22].

**Fig 3:**
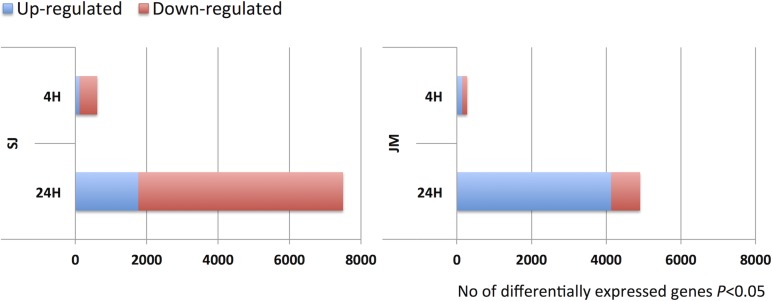
Differentially expressed genes. Number of differentially expressed genes in Sijung (SJ) and Jumli Marshi (JM) after 4 and 24h of chilling stress, respectively. Blue bars indicate up-regulated genes whereas red bars indicate down-regulated genes (BH *P*≤0.05).

**Fig 4:**
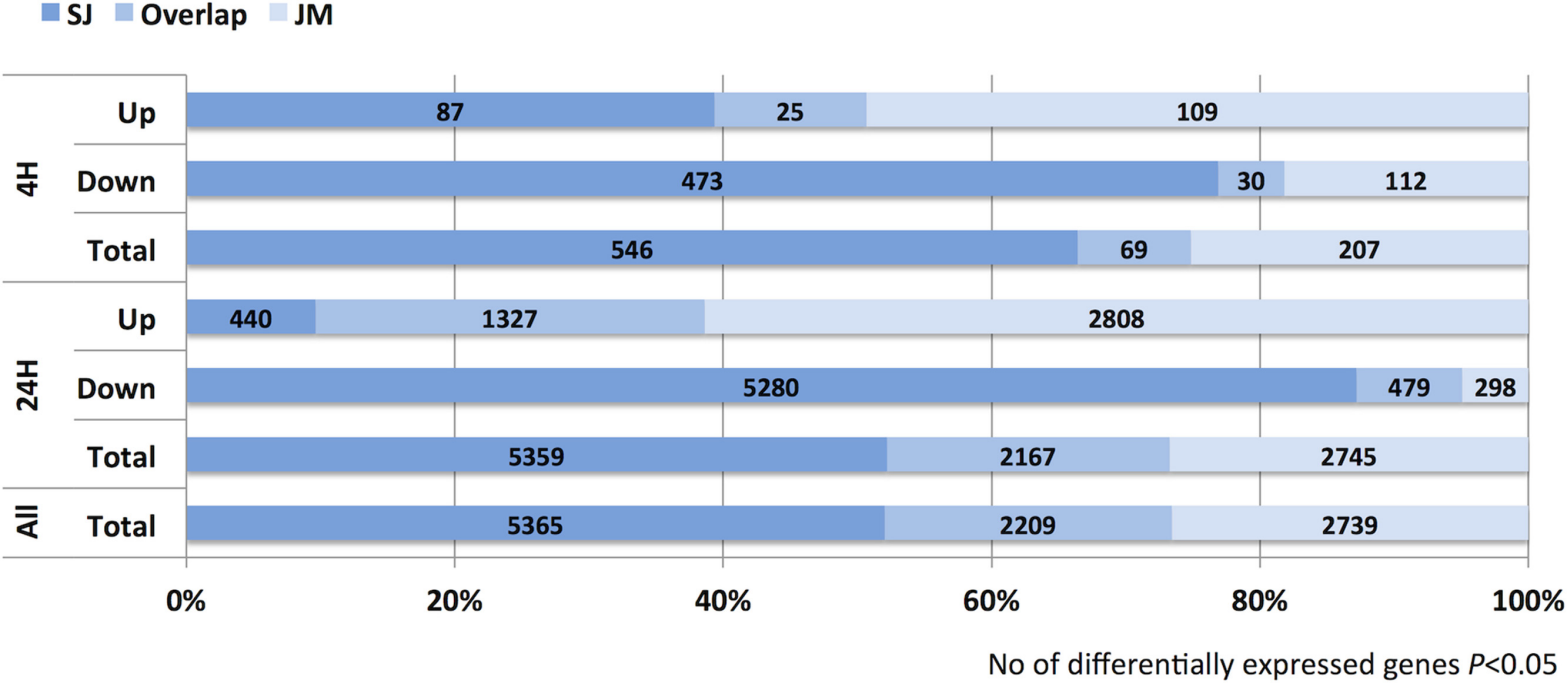
Overlap of differentially expressed genes. The figure shows the % number of genes differentially expressed (BH *P*≤0.05) in only Sijung (left dark-blue bars), in both Sijung and Jumli Marshi (middle blue bars), and in only Jumli Marshi (right light blue bars) after 4 and 24 h of chilling stress, respectively. The real number of genes for each category is written inside the bars. To note, the number of genes that overlap for Total do not up with the numbers in Up and Down, which refers to the fact that some genes are up-regulated in SJ but down-regulated in JM, and vice versa.

In total, 7,574 and 4,948 genes were found differentially expressed in SJ and JM, respectively (Fig 3). There were almost twice as many genes differentially expressed after both 4 h and 24 h of chilling stress in SJ than in JM. At 4 h, there were 570 and 231 genes differentially expressed in SJ and JM, respectively, and at 24 h these figures had increased to 7,481 and 4,867 genes, respectively. The majority of the differentially expressed genes in SJ were down-regulated (having a lower expression level compared to 0 h; 66%), whereas, in JM, they were mainly up-regulated (having a higher expression level compared to 0 h; 84%) (Fig 3). In total, 10,313 genes were found differentially expressed in either genotypes, and 2,209 (21%) of these genes were common to both SJ and JM. In more detail, number of overlapping genes in SJ and JM at 4 h were 11% and 5% of the up- and down-regulated, respectively, and at 24 h these figures were 29% and 8%, respectively (Fig 4).

Comparing the control groups of SJ and JM (i.e., time point 0 h), in total 1,721 genes were found differentially expressed. Of these genes, 30% had a strong positive correlation (PC>0.7) in expression profiles when comparing the two varieties and 34% had a strong negative correlation (PC<−0.7). The majority of the differentially expressed genes (77%) had a higher expression level in SJ compared to JM. For JM, 27% of the genes were also differentially expressed in at least one of the other time points in that variety, whereas for SJ this accounted for 73% of the genes. Nearly all of the genes (except for 5) in JM found differentially expressed in at least one of the other time points were also differentially expressed in at least one of the other time points in SJ.

### Expression profiles of chilling induced genes

Initially, to get a general overview of the gene expression changes in response to chilling stress, we analyzed the differentially expressed genes with respect to their fold change (FC) values (S1 Table). The results showed that there were more genes with at least a 5, 10 or 25 FC at 4 h in SJ than in JM; about twice as many up-regulated and three times as many down-regulated genes. At 24h, this relation was augmented regarding the down-regulated genes, however, regarding up-regulated the relation had switched and there was now more genes with a higher FC value in JM than in SJ.

**Fig 5:**
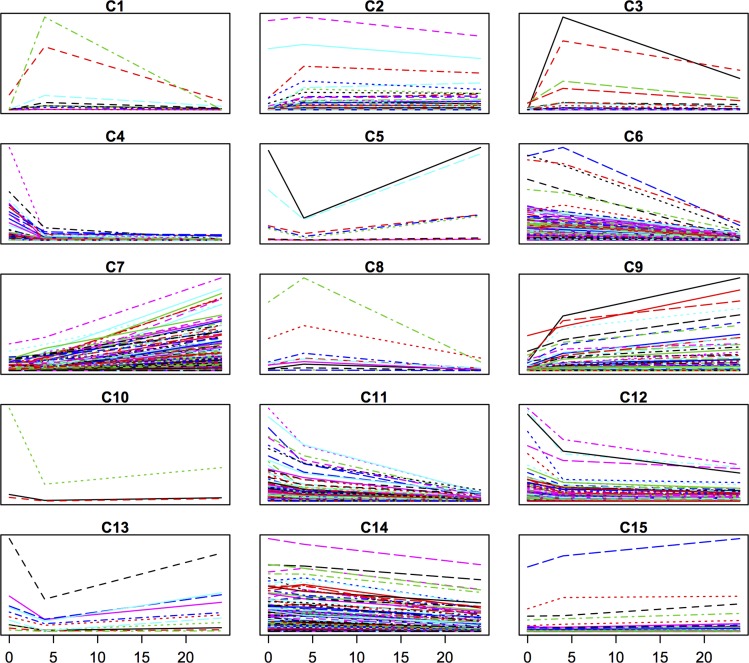
Clustering of differentially expressed genes in Sijung. Clusters produced by the STEM algorithm and based on expression data of the differentially expressed genes in Sijung; y-axis refers to gene expression levels and x-axis to time points in hours, C1–C15 refer to cluster 1 to 15, and there is one coloured line for each gene in the cluster.

**Fig 6:**
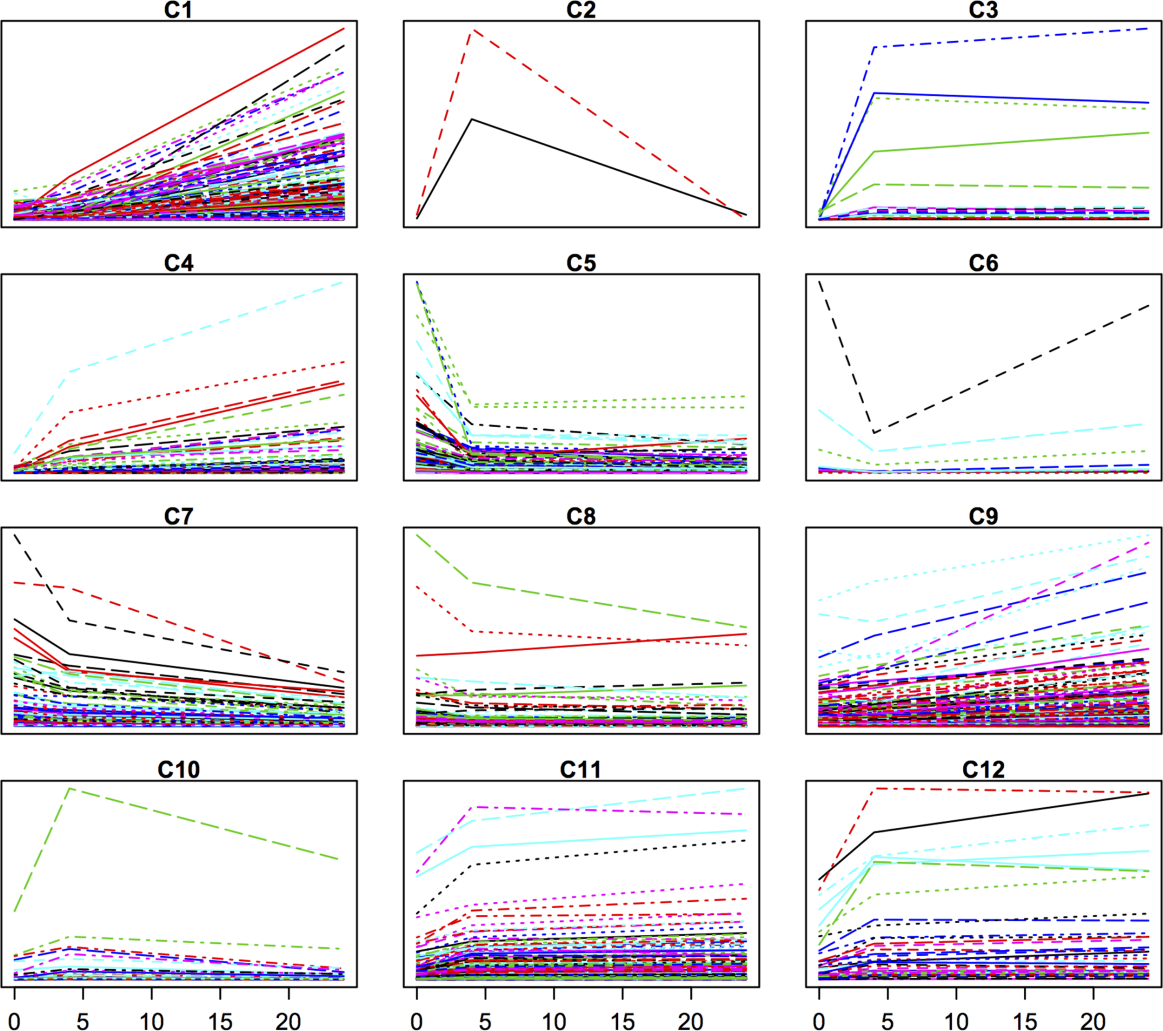
Clustering of differentially expressed genes in Jumli Marshi. Clusters produced by the STEM algorithm and based on expression data of the differentially expressed genes in Jumli Marshi; y-axis refers to gene expression levels and x-axis to time points in hours, C1–C15 refer to cluster 1 to 15, and there is one coloured line for each gene in the cluster.

Subsequently, we clustered the expression data of the differentially expressed genes for each variety separately, using the Short Time-series Expression Miner (STEM) clustering algorithm [23]. In both varieties, the majority of the responsive genes either displayed a continuous increase or decrease in expression levels over the time period studied (Figs 5 and 6). For SJ, there were six clusters (C1, C3, C5, C8, C10 and C13) out of fifteen where individual genes clearly showed a trough/peak and these in total comprised 61 genes. For JM, three clusters (C2, C6 and C10) out of twelve with such patterns could be seen, comprising in total 37 genes.

**Fig 7:**
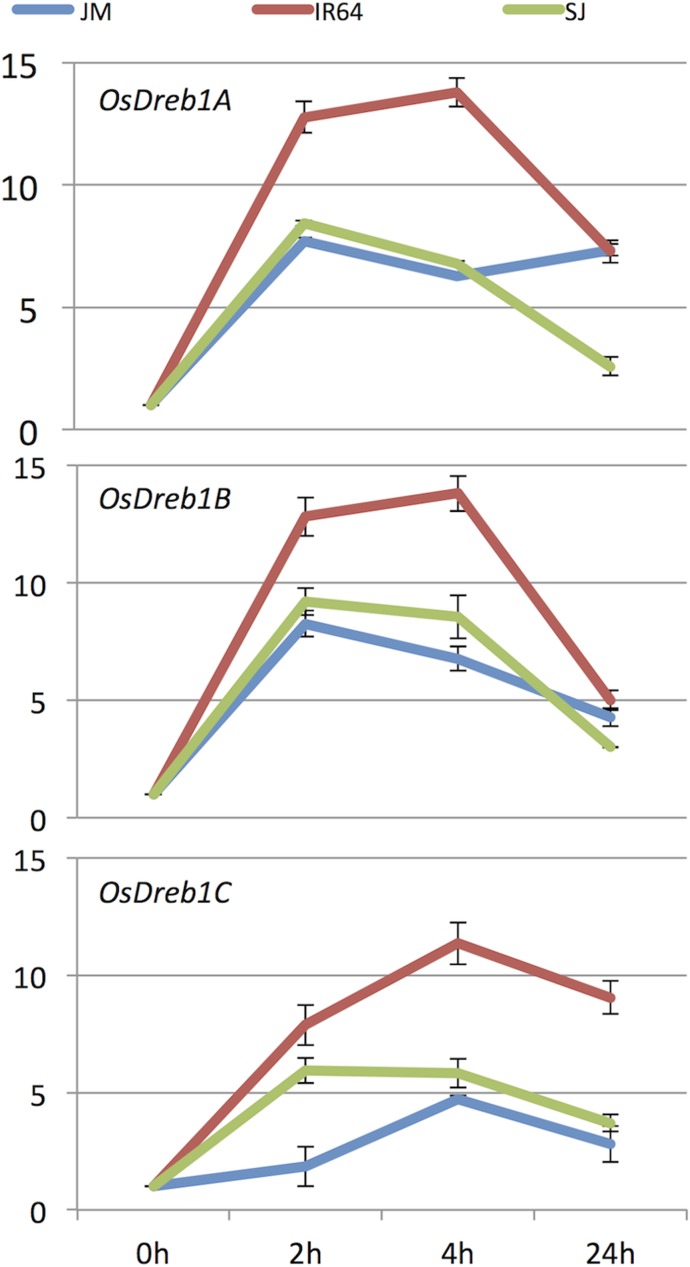
Expression of *DREB1* genes during early chilling stress. The figure shows the expression values of *OsDreb1A* (top figure), *OsDreb1B* (middle figure), and *OsDreb1C* (bottom figure) in Sijung (SJ; green line), Jumli Marshi (JM; blue line) and IR64 (IR64; red line) during the first 24 hours of chilling stress (10°C). samples were taken at four time points: 0 h, 2 h, 4 h and 24 h.

In SJ, four CBF/DREB transcription factors (TFs) were represented among those genes with a cyclic behavior (C1), but only one in JM (C6). Two of the four CBF/DREBs in SJ were the cold induced and highly upregulated, namely *OsDREB1A* and *OsDREB1B* [30, 31] and the remaining CBF/DREBs also were cold-induced but more moderately up-regulated. In contrast, in JM the CBF/DREB *OsDREB2F* displayed a deep trough during the same time period. Since the *DREB1*s have shown to be important genes in the ability to acquire chilling/cold tolerance [31–34], the mRNA expression levels of *DREB1a*, *DREB1b* and *DREB1c* were further measured by quantitative RT-PCR in SJ, JM and IR64, to reveal more subtle differences in these cultivars (Fig 7). The results show that all three genes were highly induced in all three cultivars within 4 h, but their FC levels were in general much higher in IR64 than in the others. *DREB1a* and *DREB1b* displayed the typical cyclic behavior in SJ and IR64, whereas in JM this could only be seen to a smaller extent for *DREB1b*. *DREB1c* had a flatter response pattern in all three cultivars and the response appeared to be delayed in JM (as it had a lower FC level at 2 h compared to the others).

Additionally, in total 13 of the differentially expressed genes are involved in photosynthesis; 8 were uniquely differentially expressed in SJ, 4 in JM, and one in both SJ and JM. The photosynthesis related genes in SJ were all down-regulated at 24H, whereas for those in JM one was up-regulated at 4H and the remaining up-regulated at 24H. The gene differentially expressed in both SJ and JM was Magnesium-chelatase subunit chlD, and which showed a clear opposite response pattern in the two cultivars (down-regulation in SJ and up-regulation in JM). In SJ none of the genes showed a clear cyclic behavior, whereas one gene in JM did (belonging to C10).

### Characterization of chilling induced genes

The differentially expressed genes were placed into InterPro signatures (IPRs) using the annotation data available from the Rice Annotation Project Database (RAP-DB) [24, 35]. In this way, 70% of the total 10,313 differentially expressed genes could be placed into at least one InterPro signature and in total 3,580 different signatures were represented.

**Fig 8:**
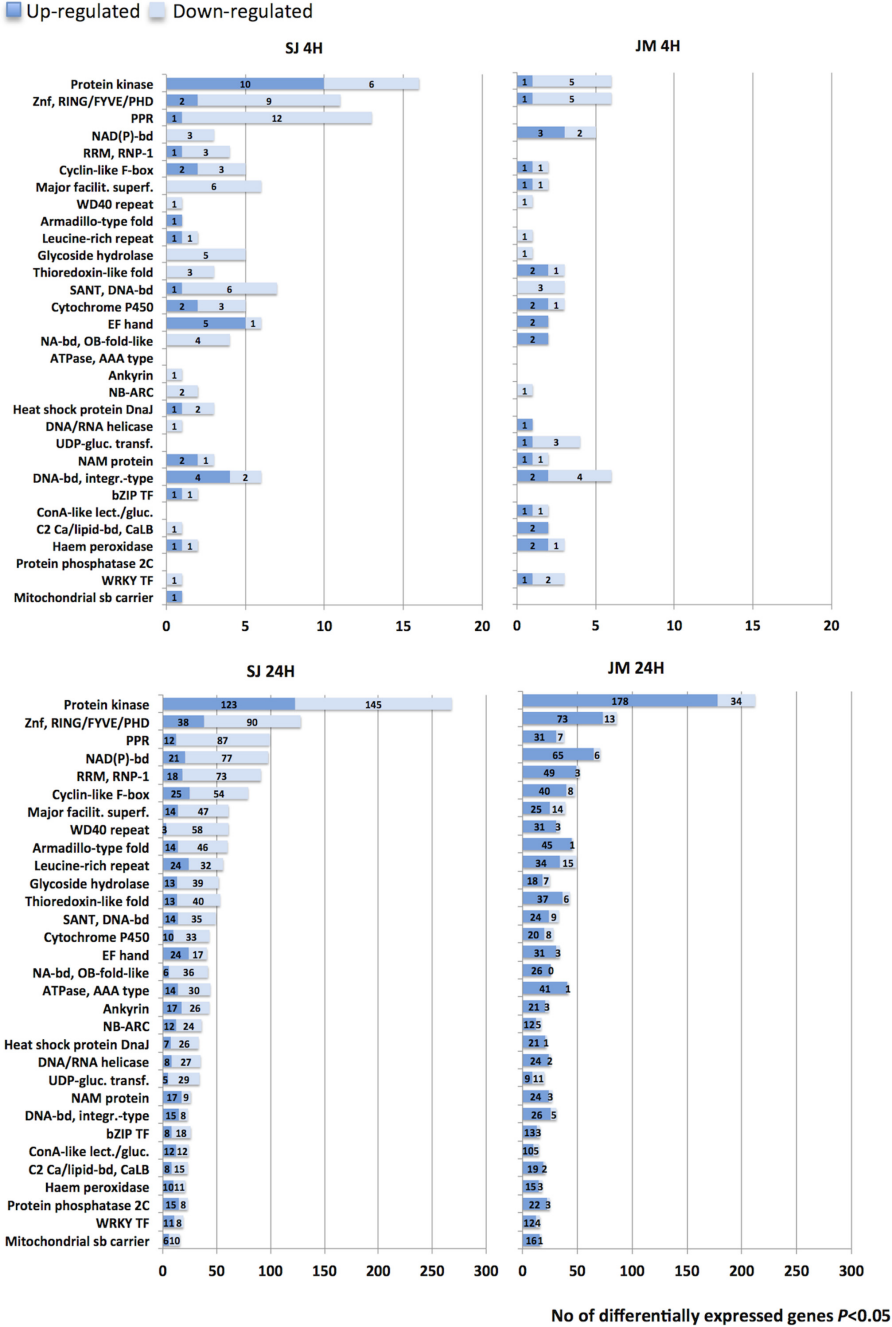
InterPro signatures. The figure show the most represented InterPro signatures among differentially expressed genes (BH *P*≤0.05) in Sijung (SJ) and Jumli Marshi (JM), respectively, at different time points. Dark blue bars indicate up-regulated genes whereas light blue bars indicate down-regulated genes.

In SJ the most frequent IPR was *Protein-kinase like* (IPR011009) at 4 h among the down-regulated (50% of these genes had been annotated with this signature). This was the same top signature in JM, but only at 24 h and among the up-regulated instead (38% of these genes). At 4 h in SJ, there was both up- and down-regulation of Protein kinases, down-regulation of Zn fingers of type RING/FYVE/PHD (Zn, RING/FYVE/PHD) and genes containing a pentatricopeptide repeat (PPR) (Fig 8). There was also up-regulation of five EF hand-containing genes, four DREB/CBF TFs (termed *DNA bd*, *integr*.*-type* in Fig 8) and down-regulation of two DREB/CBF TFs. In JM at 4 h, on the other hand, there was mainly down-regulation of protein kinases and Zn fingers of type RING/FYVE/PHD, and only two EF hand-containing genes were up-regulated. There were no PPR-containing genes differentially expressed at this time point in JM and, differing to SJ, two DREB/CBF TFs were up-regulated and four down-regulated.

At 24 h the number of up- and down-regulated had substantially increased for the majority of the IPRs (Fig 8), as the total number of differentially expressed genes had increased at this time point (Fig 3). In SJ, there were almost equally many protein kinases up- and down-regulated, whereas in JM there were substantially more up-regulated protein kinases than down-regulated. Regarding Zn fingers of type RING/FYVE/PHD and PPR-containing genes the number of down-regulated had increased substantially in SJ but not in JM. For the majority of the signatures listed in Fig 7, the same trend could be seen, i.e., in SJ there were substantially more down-regulated than up-regulated genes and vice versa in JM. However, there were a few signatures that deviated from this trend in SJ, such as the EF hand, NAM, DNA binding integrase-type (i.e., DREB/CBF and AP2 domain containing genes) and Protein phosphatase 2C signatures, for which there were more genes up-regulated.

**Fig 9:**
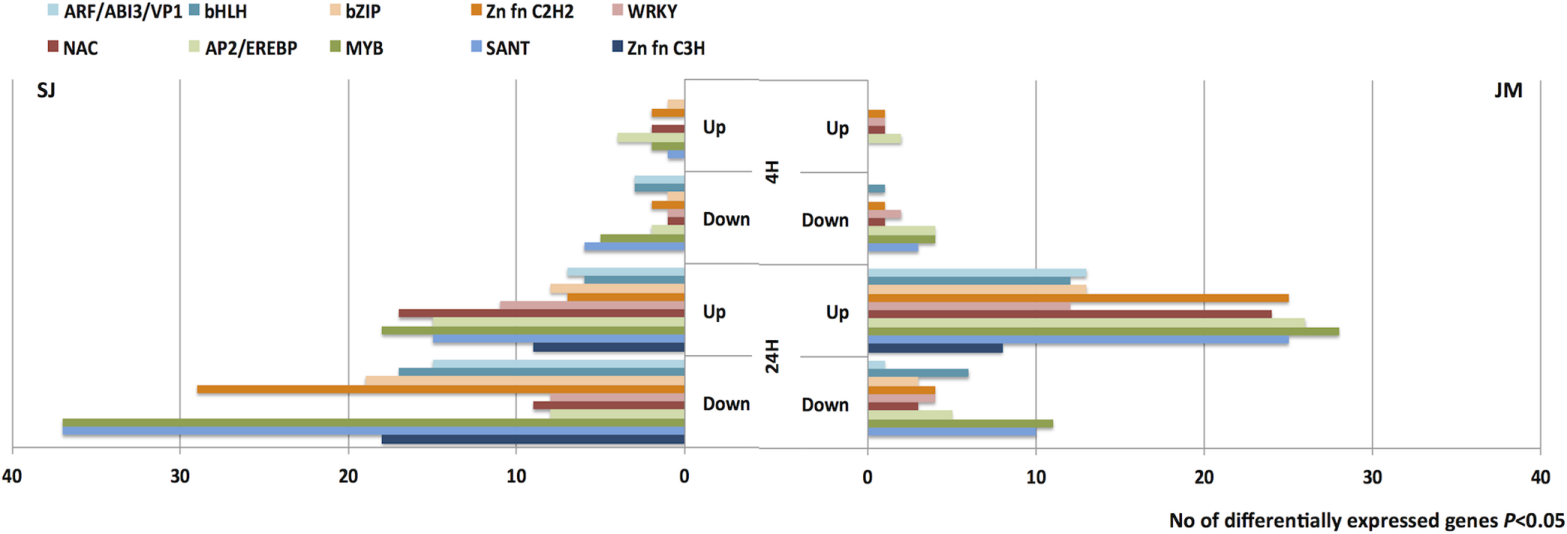
Transcription factor families. InterPro signatures were used to derive the number of TFs represented among the differentially expressed genes (BH *P*≤0.05) in Sijung (SJ) and Jumli Marshi (JM), respectively. The figure shows the most frequently represented TF families, where number of TFs for each family in SJ is shown to the left and for JM to the right.

The IPRs were subsequently used to identify TFs and their family belonging (S2 Table). In total 6% (431 genes) and 7% (326 genes) of the differentially expressed genes in SJ and JM, respectively, could be assigned to one of 39 represented TF families, and among the most frequently represented families were SANT, MYB, AP2/EREBP and NAC (Fig 9). In more detail, at 4 h there were slightly more TFs up- as well as down-regulated in SJ than in JM (14 up- and 26 down-regulated in SJ, compared to 8 up- and 20 down-regulated in JM (S1 Table), and there were only four TFs in common among the up-regulated and five among the down-regulated. At 24 h, however, this relation had changed and there were now more TFs up-regulated in JM and the contrary regarding down-regulated (145 up- and 247 down-regulated in SJ, compared to 242 up- and 62 down-regulated in JM); the number of common TFs had increased to 109 and 34 genes for up- and down-regulated, respectively. In both varieties, the most frequently occurring family among the up-regulated TFs was MYB, closely followed by AP2/EREBP. Among the down-regulated TFs in SJ the most frequently occurring families were SANT and MYB, and in JM it was MYB.

### Cellular components play distinct roles in chilling stress responses

It has previously been established that certain cellular components are highly influenced by chilling stress, such as the membranes and mitochondria [36–38]. Consequently, cellular localization annotations in combination with interesting functional annotations were derived for *up-regulated* genes in SJ and JM, respectively. This was done by using the Singular Enrichment Analysis (SEA) tool available in agriGO [25], and by this, all up-regulated genes in SJ were assigned to at least one GO term annotation, while in JM 84% of them were annotated. For SJ, 65%, 77% and 87% of the up-regulated genes had at least one Biological Process (BP), Cellular Component (CC) and Molecular Function (MF) annotation, respectively, and for JM the corresponding figures were 55%, 65% and 73%, respectively.

**Fig 10:**
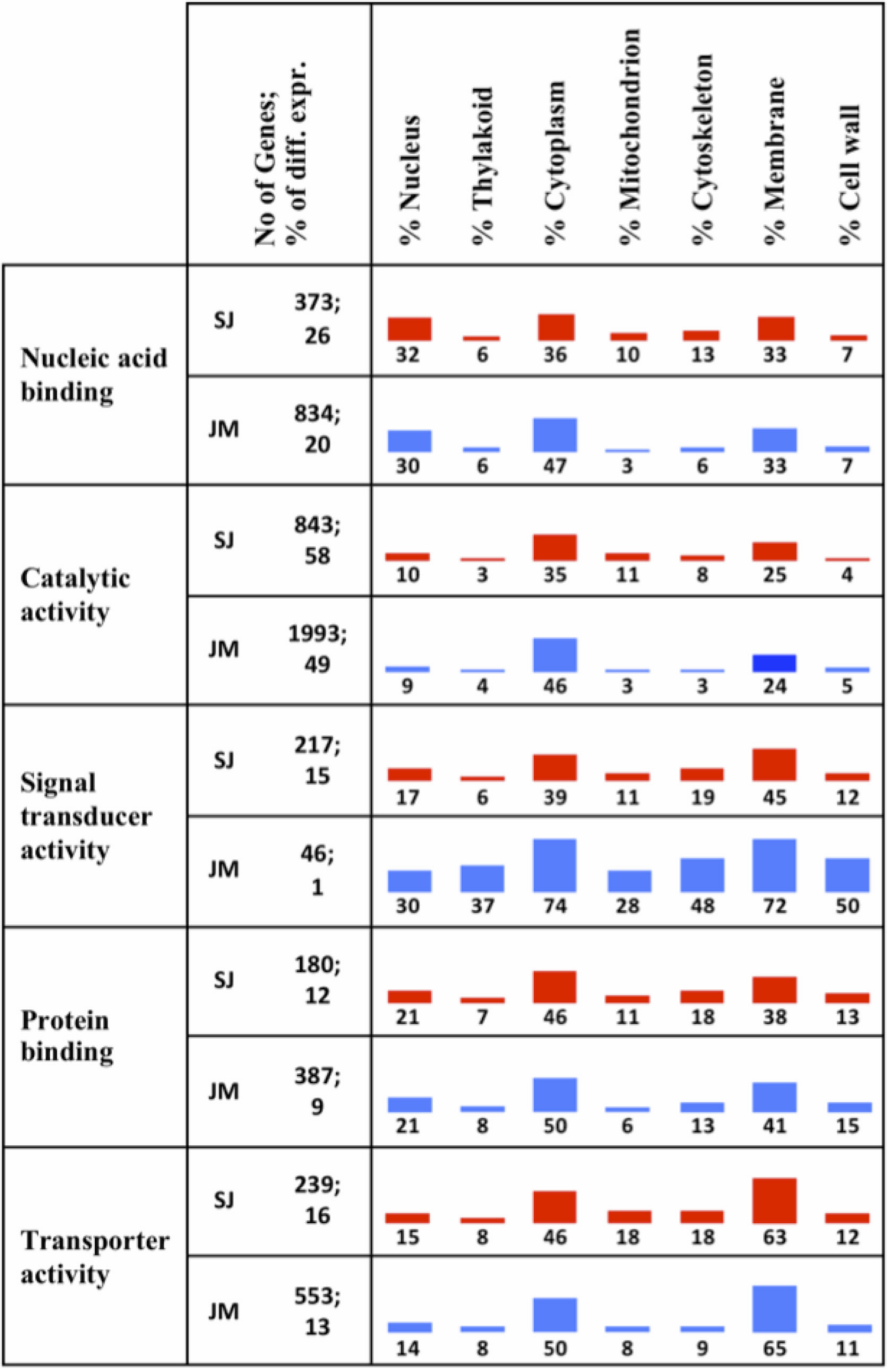
Combined view of GO annotation. The figure shows a combination of different GO molecular function and cellular component annotations. From left to right, the percentage number of genes with a specified molecular function having a product being located in any of the listed cellular components (e.g., of the 373 (26%) differentially expressed genes annotated with Nucleic acid binding in Sijung 32% have a product localized in the Nucleus).

A combined view of selected MF and CC annotations was derived, which showed the complexity of the chilling stress response in the two varieties (Fig 10). The chloroplast term was excluded, since only eight genes from SJ and none from JM could be annotated with this localization; instead the chloroplast sublocalisation term thylakoid was represented. The combined view should be interpreted from left to right and top to bottom, e.g., in SJ, 373 (26%) of the up-regulated genes had a Nucleic acid binding annotation and of those genes 32% were annotated with Nucleus, 6% with Thylakoid, 36% with Cytoplasm and so on. The results showed that for all categories listed there were about twice as many genes up-regulated in SJ than in JM (reflecting that in total about twice as many genes were up-regulated in SJ compared to JM). The only discrepancy was the term Signal transducer activity, where there were almost five times as many genes up-regulated in SJ compared to JM. Moreover, the majority of the up-regulated genes had a catalytic activity (which includes kinases, hydrolases, transferases, etc.) in both SJ (58%) and JM (49%), and overall most activities were linked to membranes and cytoplasm, and for some categories also the nucleus.

Since the GO term annotation analysis indicated that there was a discrepancy in the signal transducer activity among up-regulated genes, a more in-depth analysis of these genes was justified. Of the genes annotated with the term Signal transducer activity (STA) in SJ most of these had been annotated with the child term Receptor signaling protein serine/threonine kinase activity, whereas for JM they were mainly annotated with the term Transmembrane receptor activity (S1 Fig). Moreover, analyzing the IPRs (using previous annotation analysis) for the STA annotated genes, these were mainly related to Serine/threonine kinase, CBF/DREB/ERF TF or Zinc finger of type RING/FYVE/PHD in SJ, whereas for JM only Serine/threonine kinase-related signatures were among the most commonly occurring ones (data not shown).

### Early phase induced genes

Since the functional annotation analyses of the differentially expressed genes in the two varieties indicated differences in the underlying molecular stress responses, we decided to investigate the up-regulated genes at 4 h (UP4H) in the two varieties in more detail. The annotation of the genes reported in the databases RAP-DB and Rice Expression Profile Database (RiceXPro), as well as annotation from Arabidopsis homolog hits (as specified in RiceXPro) in the TAIR database were manually curated and summarized [24–26].

**Fig 11:**
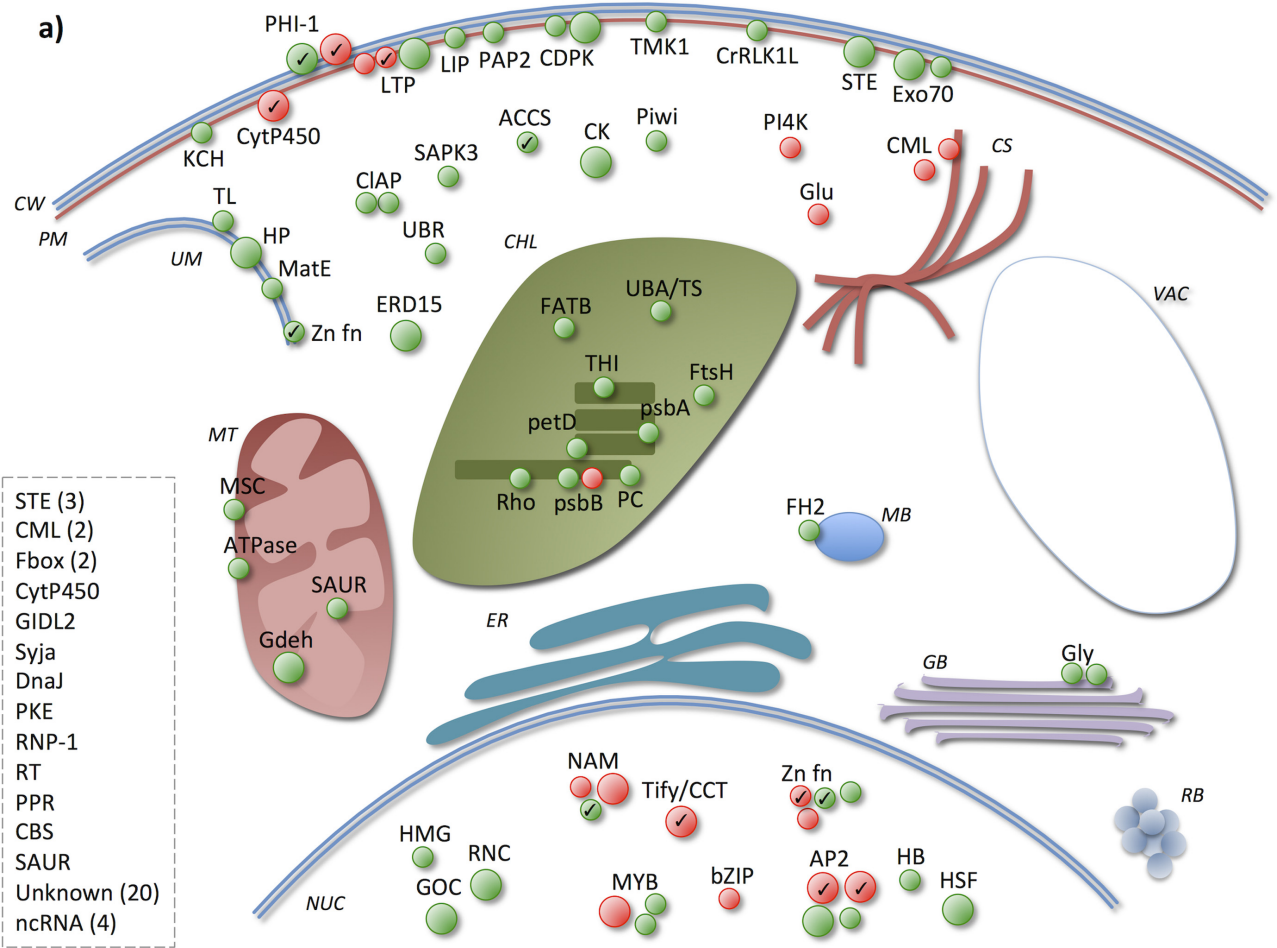
Early phase responsive genes in Sijung. The figure indicates subcellular localization of the up-regulated genes’ products at 4 h. Green and red circles indicate genes with <2,000 and >2,000 normalized microarray signal value, respectively, larger circles indicate genes with >10 FC induction, and circles with a check mark indicate genes that are also up-regulated in JM at this time point.

**Fig 12:**
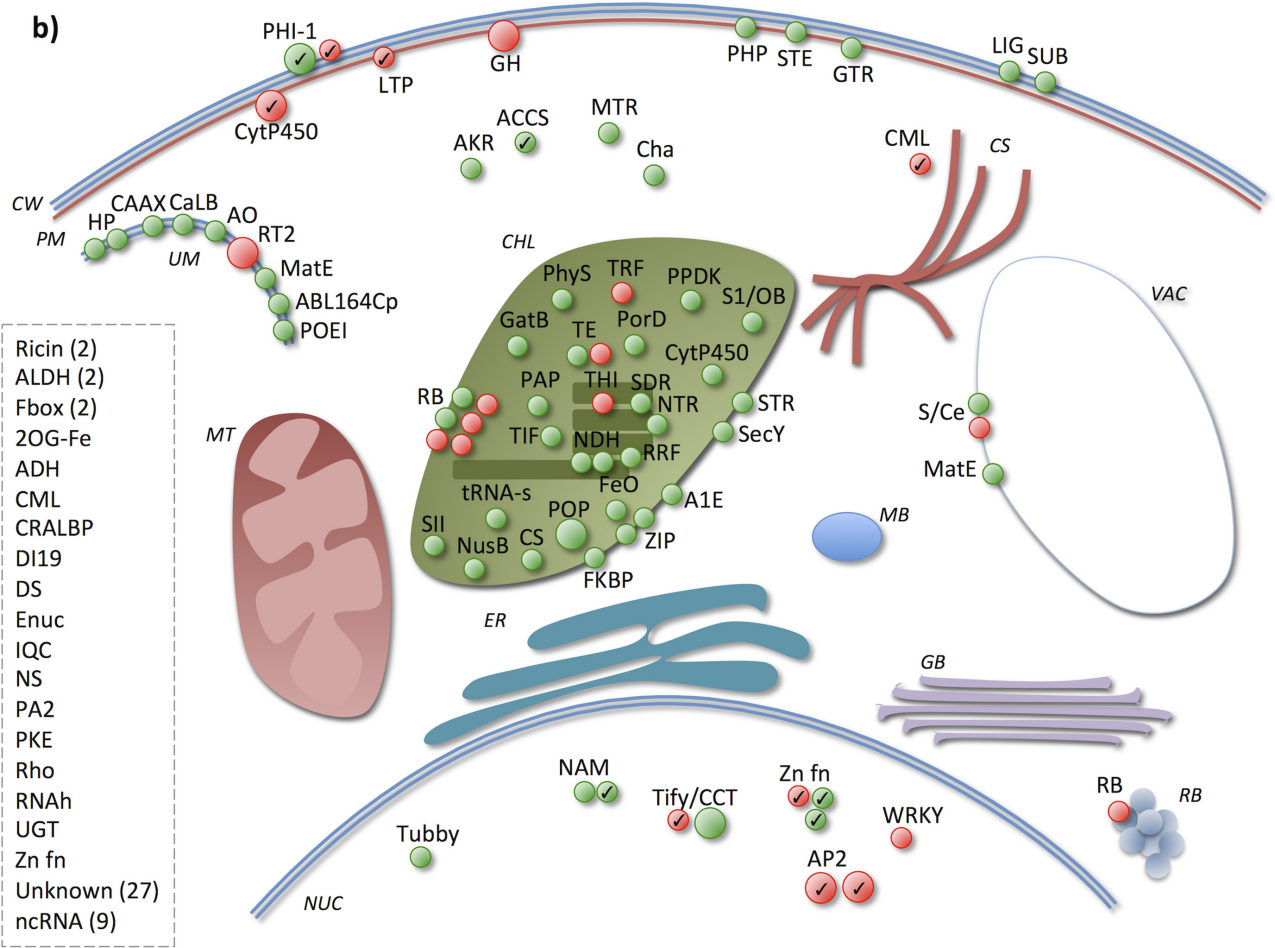
Early phase responsive genes in Jumli Marshi. The figure indicates subcellular localization of the up-regulated genes’ products at 4 h. Green and red circles indicate genes with <2,000 and >2,000 normalized microarray signal value, respectively, larger circles indicate genes with >10 FC induction, and circles with a check mark indicate genes that are also up-regulated in SJ at this time point.

Here, focus was on where in the cell different processes take place, by studying the gene products’ subcellular localization in combination with their molecular function and the biological process(es) they participate in (S3 Table). In total, 63% and 56% of the UP4H in SJ and JM, respectively, had an annotated subcellular localization, and 76% and 75% of the UP4H in SJ and JM, respectively, had a molecular function and/or biological process annotation. Overall, of those products with a subcellular localization, both in SJ and JM these were mainly annotated with membrane, of which the plasma membrane (PM) was most represented in SJ and chloroplast membrane (CM) in JM (Figs 11 and 12). Regarding PM, there were more genes annotated with this localization in SJ than in JM among the UP4H. Second most represented localization annotation was the *nucleus* for SJ and chloroplast for JM. Next, a more detailed description of the UP4H will be given, however, since it is not possible to cover all genes, only some of them will be highlighted here.

In both varieties, two genes with cell wall (CW) localization annotation were induced and both of these contain a conserved region previously identified in a phosphate-induced gene (PHI-1) (Figs 11 and 12); the function of this region is, however, still unknown [39]. In SJ, both of these genes were up-regulated by >10-fold, whereas, in JM, only one of them was >10-fold induced. Phosphate (P) is important for a range of biological functions, e.g., being a structural element in phospholipids (which, in turn, are major components of cell membranes), involved in signal transduction and in the regulation of enzymes [40]. Interestingly, P deprivation stress has previously been shown to be beneficial for cold acclimation [41].

Regarding PM, a cytochrome P450 (CytP450) localized to the plasma membrane (PM) was highly induced in both varieties (>10-fold up-regulated). This gene belongs to the cytochrome family 707 subfamily A and its Arabidopsis orthologs encode ABA 8'-hydroxylases that are prominent in the catabolism of abscisic acid (ABA), participate in oxidation reduction processes, regulate stomatal movement and react to red/far red light [42–46]. There was also a lipid transfer protein (LTP) induced in JM annotated with this localization, whereas in SJ three such genes were induced. Plant LTPs are, amongst others, important in the biosynthesis and transportation of cutin, which is the main component of the plant cuticle and essential for the cuticle to function as a barrier against water loss [47, 48]. In SJ there was also an induction of two calcium-dependent serine/threonine protein kinases (CDPK) and of which one was >10-fold induced. In JM, on the other hand, no CDPKs with a PM localization annotation were activated at this time point. CDPKs participate in protein phosphorylation and ABA-mediated signaling, function as calcium sensors and participate in the regulation of stomatal movement [49]. They have also been implicated in various plant stress responses [50–52]. In SJ, there was also three additional protein kinases (STE, TMK1 and CrRLK1L) activated, which have previously been shown to be involved in protein phosphorylation, signal transduction and abiotic stress responses [49, 53–55].

Other genes induced by chilling stress in SJ with a PM localization annotation were Exo70 genes, which products constitute a subunit in the exocyst complex and participate in the late stage of exocytosis [56], as well as a lipase (LIP), which product hydrolyses lipids [57], and a lipid phosphatase (PAP2), which product participates in the synthesis of phospholipids and lipid signaling [58]. These genes are possibly involved in the transportation of lipids and other substrates via exocytosis as a response to PM disruption caused by the chilling stress [36, 56, 59, 60]. Regarding JM, on the other hand, there was a xyloglucan endotransglucosylase/hydrolase (GH) that was highly induced and which has previously been shown to participate in cell wall loosening and in the protection of the cell against dehydration [61, 62]. There was also a phosphate permease (PHP) previously shown to transport inorganic P and that might be coupled to P deprivation stress during cold stress [41, 63], a serine/threonine/tyrosine protein kinase (STE), which is coupled to phosphorylation and signal transduction during abiotic stress [49, 53–55] and a glutathione S-transferase (GTR), which possibly participates in oxidation reduction and detoxification of toxic substrates that accumulates during abiotic stress [64].

In JM the chloroplast seemed to be highly activated very early, since a large number of the up-regulated gene products had a chloroplast/thylakoid localization annotation, whereas there seemed to be less chloroplast activity in SJ as fewer genes annotated with chloroplast localization were induced. In JM, 12 of these genes could be coupled to protein synthesis; two translation elongation factors (TE), six ribosomal proteins (RB), one involved in the biosynthesis of tRNA (tRNA-s), a peptidyl-propyl isomerase (FKBP), a ribosome recycling factor (RRF), and a prolyl oligopeptidase (POP) with a role in the modification of proteins and peptide degradation (such as hormones) [64–67]. Intact chloroplast translation has previously been shown to be important for cold/abiotic stress tolerance [68–70]. Regarding SJ, only four of the UP4H could be coupled to the photosystem: cytochrome b_6_f subunit (petD), photosystem II P680 chlorophyll A apoprotein (psbB), photosystem II psbA (psbA) and a plastocyanin-like domain containing (PC) [71, 72]. However, none of these genes were highly up-regulated (i.e., <10-fold). Previous studies have shown that photosystem II is inhibited in sensitive species during chilling/cold stress and is a part of their sensitivity [73, 74]; however, the activation of genes coupled to the photosystem indicate that it was functional during the stress attributed here.

In SJ there were substantially more genes induced which product was annotated with cytoplasm localization compared to JM. For example, in SJ there were two calcium-binding EF-hands (CML) induced, but only one in JM. These genes function as calcium sensors and are involved in calcium dependent signaling. Stress responses in plants are critical dependent on calcium signaling transduction pathways, and sensor molecules such as calcium binding proteins commonly perceive changes in calcium levels and activate different response pathways [75].

Regarding nucleus localization, there were more TFs induced in SJ than in JM. Interesting to note is that there were a number of TFs highly induced in SJ but not in JM, namely, a NAM (NAC), a MYB, two AP2/EREBPs and a Tify/CCT. In JM, on the other hand, there was a WRKY TF induced at this time point, but not in SJ. TFs of these families have previously been shown to be key regulators of various abiotic stress responses in plants [76]. The Tify/CCT and WRKY TFs expressed in JM could plausibly be coupled to the activation of the jasmonic acid (JA) pathway as a response to wounding [77, 78]. Moreover, there was a phospholipase A2 (PA2) with an unknown subcellular localization highly up-regulated at this time point in JM, which could also possibly be linked to this signaling pathway [79].

### Physiological evaluation of cold tolerance

**Fig 13:**
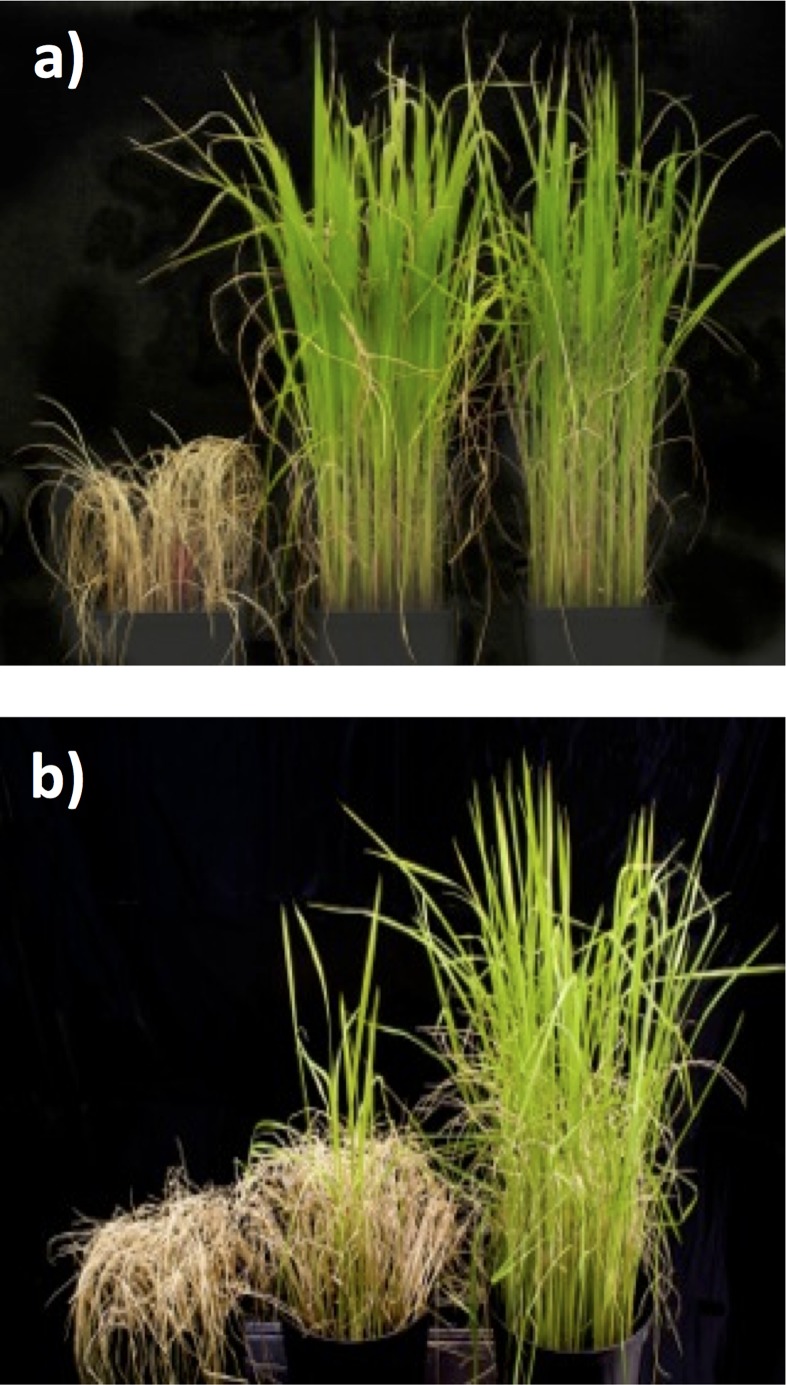
Plants exposed to cold stress. Seedlings of IR64, Jumli Marshi and Sijung (from left to right) were grown for three weeks under regular growth conditions (a) and then moved to chilling conditions (4°C) for seven days and thereafter allowed to recover for ten days (b).

Since our initial chilling stress studies showed that SJ is slightly more tolerant than JM, and apparent differences in the underlying molecular responses could be seen, we explored their tolerance to cold stress at 4°C. While we are mainly interested in chilling stress in this work, to be able to visually see the difference in the survival of two the chilling tolerant rice lines SJ and JM, cold stress conditions are used for evaluation. Physiological responses were evaluated and compared between the two varieties by exposing three weeks old seedlings to cold conditions (4°C) for seven days and thereafter allowing them to recover for ten days in regular growth conditions. The results showed that IR64 was most sensitive to cold stress as the seedlings were killed after seven days at 4°C, but JM and SJ seedlings still showed viability (Fig 13). Additionally, SJ appeared to be more tolerant than JM as its recovery was better after the stress treatment compared to JM (Fig 13).

## Discussion

Rice yield is severely limited by various abiotic stresses, low temperatures being one of them, and breeding for more tolerant varieties is a necessity to increase the productivity. However, to promote the development of such varieties more knowledge about the underlying molecular responses and, importantly, dissimilarities in these responses among rice varieties would be helpful. It has previously been established that underlying molecular differences in genotypes of a common species with contrasting cold/chilling tolerance levels exist (e.g., [15, 80–85]). In rice, such differences have also been identified [86, 87], and, interestingly, also in genotypes with similar tolerance levels [88]. We contribute to the latter field by this study, since the results clearly imply that differential, but overlapping, response strategies are undertaken by the varieties Sijung and Jumli Marshi, which both are relatively tolerant to chilling stress, albeit at different levels.

In broad GO annotation terms, the activated response pathways in SJ and JM seemed to be coherent, as the terms were consistent for the up-regulated genes in the two varieties. The majority of the genes had a catalytic, transcription regulator or transporter activity, and their products were mainly concentrated to the cytoplasm and membranes. The only discrepancy seemed to be signal transducer activity, where substantially more genes were induced in SJ than JM. Many genes previously coupled to chilling and/or cold stress in rice and other related species were among the differentially expressed also in SJ than JM, e.g., TFs like AP2/EREBP, MYB and NAC, Serine/threonine protein kinases and EF-hand containing genes [49, 50, 75, 89]. Chilling/cold stress commonly manifests in a decrease in photosynthesis, increase of reactive oxygen species (ROS), over-reduction of the chloroplast electron transport chain and damages to membrane integrity, which ultimately lead to cellular dehydration, osmotic imbalance and other deleterious effects [1, 89]. More tolerant species have developed responses to cope with this, such as changes in membrane composition, increase of soluble sugars and production of ROS scavengers.

Previous research has shown that circadian rhythms influence photosynthetic processes and play an important role in coordinating photosynthetic activity with the diurnal changes in light, and that chilling temperatures disrupt or stall photosynthesis processes in warm-climate plants [90–93]. The microarray contains 38 rice genes annotated with the GO term photosynthesis and a few of these were differentially expressed during the chilling treatment. Indeed, only 8 genes in SJ and 5 in JM related to photosynthesis were differentially expressed, and moreover, only one gene in JM displayed a clear cyclic behavior (which indicates preserved circadian regulation). This follows previous finding, that photosynthesis processes are affected by chilling temperatures.

In both varieties, there was an activation of genes encoding lipid transfer proteins as well as genes involved in sugar production pathways, such as dehydrogenases/reductases and NAD-dependent epimerase/dehydratases, which can be coupled to membrane- and osmoprotection [1, 47, 48, 89]. Regarding ROS scavengers [1, 89], heme peroxidases and glutaredoxins/thioredoxins were most abundant among the differentially expressed genes in both varieties, although catalases, glutathione S-transferases, ascorbate peroxidase and superoxide dismutates were also represented.

On the other hand, the most abundant molecular function gene groups, calcium and phosphorylation signaling pathways were after 4 h in SJ mainly represented by up-regulated genes like serine/threonine and/or tyrosine kinases and calcium-binding EF-hands. The kinases and calcium-binding EF-hands were also top abundant at 24 h, but were also accompanied with genes coupled to ubiquitination (zinc finger RING-type and cyclin-like F-box), post-transcriptional regulation (RNA recognition motif) and sugar production (epimerases/dehydratases). Regarding JM at 4 h, the responses were more scattered on different functions/pathways, with generally less number of up-regulated genes in each group. The top abundant gene groups were coupled to sugar production (epimerases/dehydratases), detoxification (multi antimicrobial extrusion proteins and aldehyde dehydrogenases), ROS scavenging (glutaredoxins/thioredoxins, peroxidases and glutathione S-transferases), translation (translation elongation factors and ribosomes), signaling (calcium-dependent EF-hands and calcium-dependent phospholipid binding membrane targeting) and plausibly the activation of the jasmonic acid pathway (Tify/CCT, PA2, WRKY). At 24 h the most abundant gene groups are very much similar to those in SJ, i.e., signaling (protein kinases and calcium-dependent EF-hands), ubiquitination (zinc finger RING-type and cyclin-like F-box), post-transcriptional regulation (RNA recognition motif) and sugar production (epimerases/dehydratases), but are also accompanied with transportation (ABC transporters) and ROS scavenging (glutaredoxins/thioredoxins, peroxidases and glutathione S-transferases).

To note, at 4 h there were more activity in the plasma membrane in SJ than in JM and the contrary regarding the chloroplast. In SJ, there were also several genes up-regulated which encode proteins coupled to signal transduction, lipid transport and exocytosis, and which could not be seen in JM. Some of the signal transduction proteins were calcium-dependent protein kinases, which may be involved in the regulation of calcium-dependent exocytosis and stomatal closure in response to the chilling stress [49, 94–96]. The lipid transfer proteins most likely transport cutin or wax to the plasma membrane as a protection against water loss and the exocytosis is plausibly activated as a response to plasma membrane disruption caused by the stress [59, 97]. Chloroplast translation has previously been shown to be important for cold/abiotic stress tolerance [68–70] and several genes encoding proteins coupled to this process were up-regulated in JM, such as ribosomal-, translation elongation factor- and ribosome recycling proteins, which was not the case in SJ.

A key initial event that occurs in the cold stress response is the induction of CBF/DREB TFs, which belong to the AP2/EREBP TF family, and they are commonly induced within 30 minutes of cold treatment in plants [7, 10, 11, 30, 31, 33, 34, 98]. In this study, *DREB1A* and *DREB1B* were highly induced in both SJ and JM within 4 h, but *DREB1C* showed only a slight induction in JM. On the other hand, SJ displayed a higher level of stress tolerance against chilling than JM, which indicates that part of the differences lie in the activation of other response pathways. The CBF/DREB genes have also been shown to be gated by a circadian clock and display a cyclic behavior during cold stress [33, 34, 98]. For *OsDREB1A* and *OsDREB1B* in SJ during chilling stress this behavior is re-confirmed, however, it seems to be over-ridden in JM since, although the genes were highly up-regulated, the expression levels were nearly constant after 4 h of stress (data not shown).

Finally, a lesser decrease in *F*_v_/*F*_m_ ratio in combination with more genes differentially expressed and also with higher FC values at 4 h in SJ, indicates that a more rapid response, and plausibly a better adjustment, to the stress takes place in SJ than in JM. The reason seems to be a more efficient activation of genes during the stress response that encode products participating in protecting the plasma membrane and at the same time an inactivation of non-participating genes.

## Conclusion

In this study, we conducted global microarray analyses to identify genes induced during early chilling stress in the two rice varieties Sijung and Jumli Marshi, both chilling tolerant, but to different levels. Our analyses showed that both varieties share response pathways with temperate plants, as many previously identified cold responsive genes from other plant systems were also induced in both SJ and JM. Additionally, the study showed that different strategies to handle the stress are undertaken by the two varieties, since although to some degree overlapping, differential underlying molecular responses were induced. Further investigation and validation of these identified similarities and differences may lead to the development of new molecular markers to be used in the breeding for more chilling tolerant rice varieties.

## Supporting Information

S1 Fig Up-regulated genes in SJ and JM that have been annotated with the GO term Signal transducer activity. (TIFF)

S1 Table Fold change (FC) values of differentially expressed at different time points and for each variety. (PPTX)

S2 Table InterPro signatures relating to TF families and the number of differentially expressed genes containing respective signature. (XLSX)

S3 Table Subcellular localization of early phase responsive genes, i.e., genes up-regulated at 4 h. (XLSX)
