# Supplementary figures and images for: Comparative Transcriptomics of Sijung and Jumli Marshi Rice during Early Chilling Stress Imply Multiple Protective Mechanisms
Source: PLoS One. 2015 May 14;10(5):e0125385. doi: 10.1371/journal.pone.0125385 (PMC4431715; doi:10.1371/journal.pone.0125385)

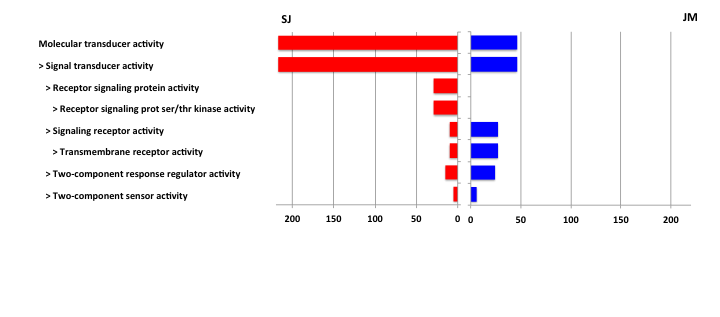

Supplement: S1 Fig — (TIFF) [file pone.0125385.s001.tiff]
