# Supplementary material for: Comparative Transcriptomics of Sijung and Jumli Marshi Rice during Early Chilling Stress Imply Multiple Protective Mechanisms
Source: PLoS One. 2015 May 14;10(5):e0125385. doi: 10.1371/journal.pone.0125385 (PMC4431715; doi:10.1371/journal.pone.0125385)
Supplement: S1 Table — (PPTX) [file pone.0125385.s002.pptx]

## Slide 1
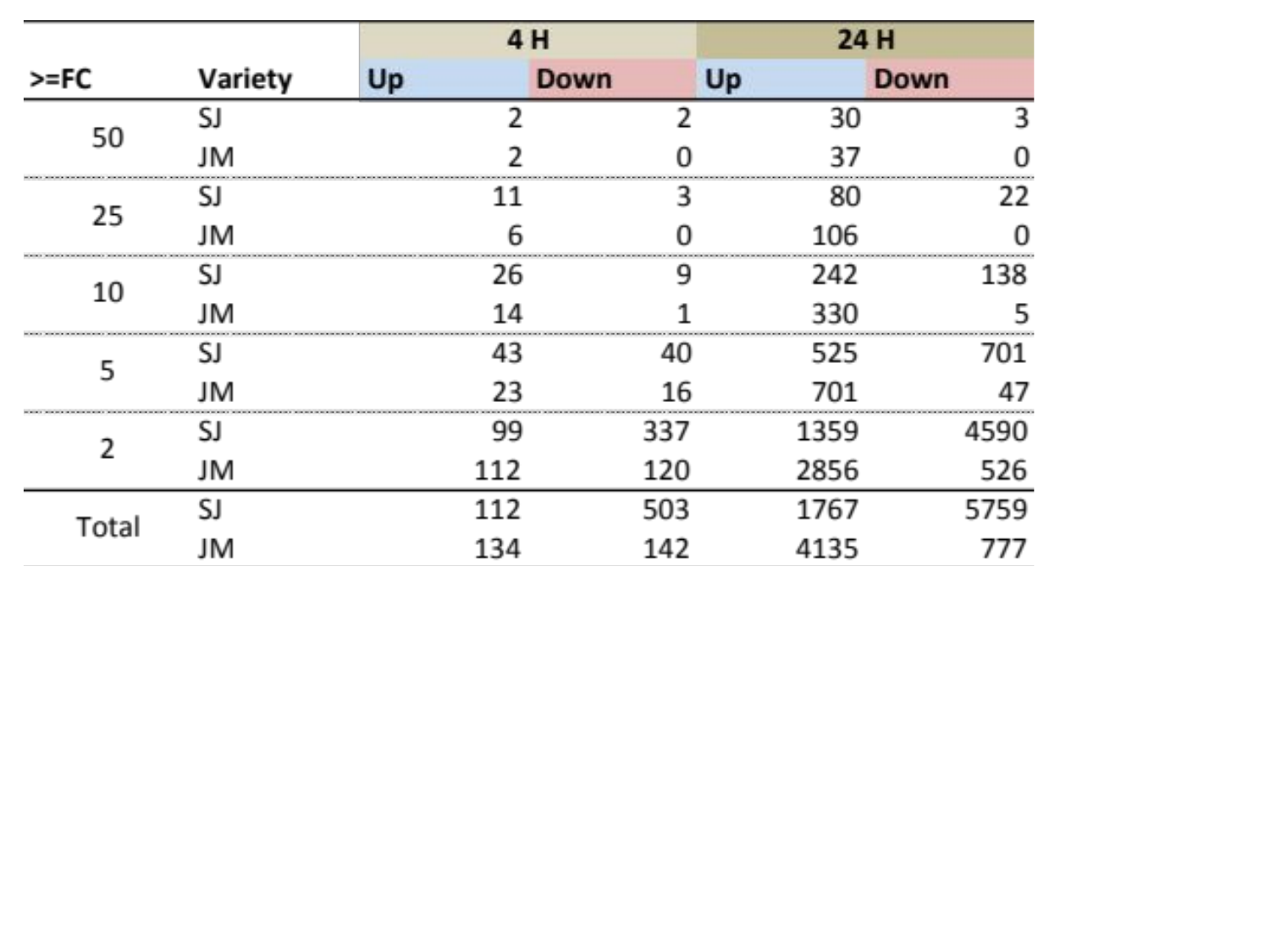

## Slide 2
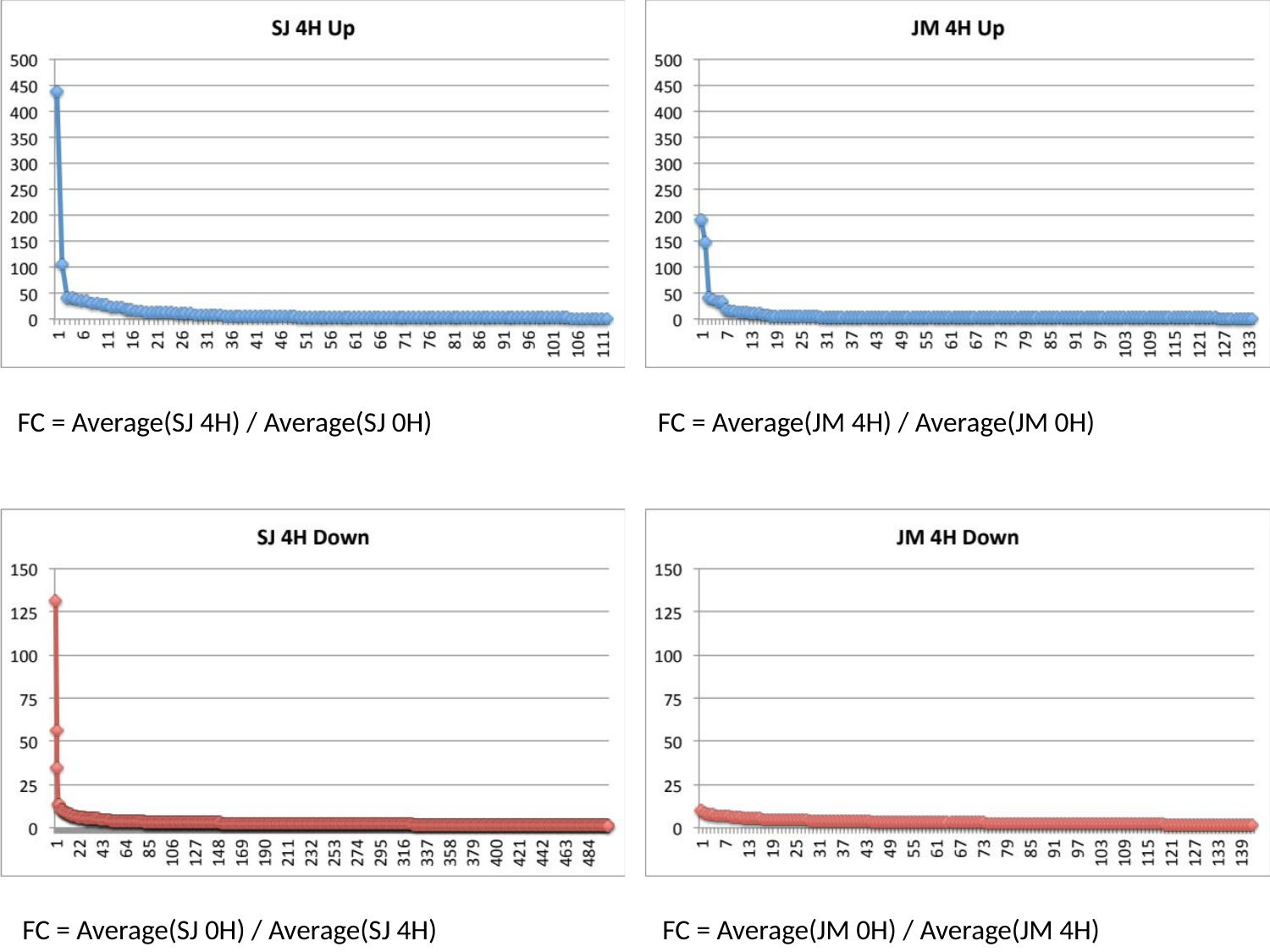

FC = Average(SJ 4H) / Average(SJ 0H)
FC = Average(JM 4H) / Average(JM 0H)
FC = Average(SJ 0H) / Average(SJ 4H)
FC = Average(JM 0H) / Average(JM 4H)

## Slide 3
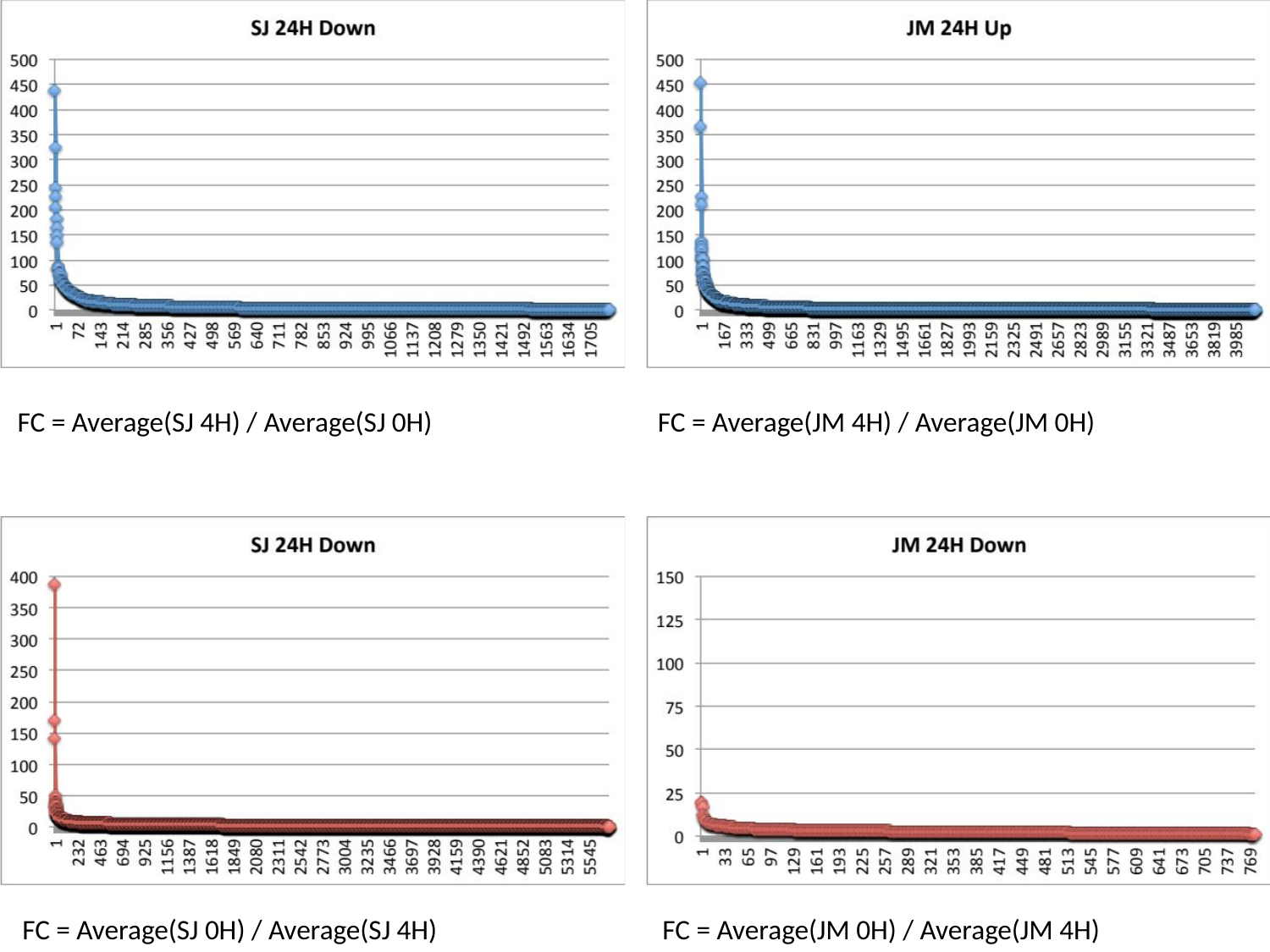

FC = Average(SJ 4H) / Average(SJ 0H)
FC = Average(JM 4H) / Average(JM 0H)
FC = Average(SJ 0H) / Average(SJ 4H)
FC = Average(JM 0H) / Average(JM 4H)
